# Efficacy of Different Techniques of the Inferior Alveolar Nerve Block for Mandibular Anesthesia: A Comparative Prospective Study

**DOI:** 10.7759/cureus.53277

**Published:** 2024-01-31

**Authors:** Sai Krishna, Kathiravan Selvarasu, Santhosh P Kumar, Murugesan Krishnan

**Affiliations:** 1 Oral and Maxillofacial Surgery, Saveetha Dental College and Hospitals, Saveetha Institute of Medical and Technical Sciences, Saveetha University, Chennai, IND

**Keywords:** fischer 1-2-3 technique, gow-gates technique, vazirani-akinosi technique, kurt-thoma technique, inferior alveolar nerve block, innovative technique, novel therapies, mandibular anesthesia, pain perception, conventional halsted technique

## Abstract

Background

The inferior alveolar nerve block (IANB) is a commonly employed technique in oral surgery for achieving profound anesthesia in the mandibular teeth and associated structures. Several techniques have been developed to enhance the success rate and patient comfort during the IANB. The aim of this study was to compare and evaluate the efficacy of different IANB techniques for mandibular anesthesia.

Materials and methods

The participants included in the study were adults requiring surgical extraction of an impacted mandibular third molar teeth. A total of 100 participants were randomly assigned to five different groups representing various techniques of IANB, i.e., conventional Halsted technique, Vazirani-Akinosi technique, Gow-Gates technique, Fischer 1-2-3, and extraoral Kurt-Thoma technique, with 20 participants in each group. The participants were evaluated for the onset of anesthesia using subjective and objective methods, pain perception during the administration of local anesthesia using a 10-point visual analogue scale (VAS), and the incidence of trismus postoperatively. Data were analyzed using IBM SPSS Statistics for windows, version 23.0 (released 2015; IBM Corp Armonk, United States) with p-values less than 0.05 considered as statistically significant. Descriptive statistics, Kruskal-Wallis, and post-hoc tests were included in the data analysis for intergroup comparisons.

Results

The primary outcomes evaluated were the onset of anesthesia, the patient's perception of pain during the administration of local anesthesia, and the secondary outcome included in the incidence of trismus. In this study, it was found that the Kurt-Thoma technique had the fastest onset of anesthesia (2.25 minutes), higher incidence of trismus (25%), and higher pain perception (6.5 score on VAS). The conventional Halstead technique (3.55 minutes), Fischer 1-2-3 technique (3.5 minutes), and Vazirani-Akinosi technique (3.1 minutes) had an almost similar mean duration of anesthesia. The onset of anesthesia was delayed in the Gow-Gates technique (5.1 minutes). Patient perception of pain during administration of local anesthesia was higher in the Kurt-Thoma (6.5) and Gow-Gates techniques (4.95), and it was least in the Fischer 1-2-3 technique (0.75) in the VAS scores. The incidence of trismus was highest with the Kurt-Thoma technique (25%), then the Gow-Gates technique (20%), followed by the conventional Halstead technique (5%).

Conclusion

In this study, it was found that the conventional Halsted technique was the best among the different techniques of IANB and remains the gold standard.

## Introduction

Local anesthesia is an essential component of dental procedures providing pain control and ensuring patient comfort during various dental treatments [1]. The inferior alveolar nerve block (IANB) is widely employed due to its ability to provide profound anesthesia to the mandibular teeth and associated structures [2]. Various techniques have been practiced to enhance the speed of onset anesthesia and to increase the success rate of the IANB. These techniques include the conventional Halsted technique, Vazirani-Akinosi technique, Gow-gates technique, Fischer 1-2-3 technique, and extraoral technique, i.e., Kurt-Thoma technique [3]. The duration of the onset of anesthesia is a critical parameter in dental procedures as it directly affects treatment efficiency [4]. By comparing the onset of anesthesia among these techniques, the most effective method for achieving rapid anesthesia can be identified.

Pain during local anesthesia injection has been a significant concern for dental patients, and minimizing this discomfort is a crucial factor affecting patient satisfaction and thereby providing optimal care [5]. Pain perception during injection was assessed in the published literature using a 10-point visual analog scale (VAS) to determine the least painful injection experience [4-6]. Trismus or restricted mouth opening is a potential complication associated with IANB techniques [7].

The aim of the study was to assess the efficacy of different techniques of the IANB for mandibular anesthesia. The primary objectives of this study were to compare the onset of anesthesia and patient perception of pain during administration of a local anesthetic using different techniques of IANB. The secondary objective was to assess the incidence of trismus among different IANB techniques. This study will improve clinical practice regarding dental anesthesia by providing evidence-based recommendations for the selection of IANB techniques. Identifying the most efficient and patient-friendly technique will enhance treatment outcomes, reduce patient anxiety, and improve overall patient satisfaction.

## Materials and methods

Study design and setting

This prospective study was carried out in the Department of Oral and Maxillofacial Surgery, Saveetha Dental College and Hospitals, Chennai, India. The study protocol was approved by the Institutional Human Ethics Committee of Saveetha Dental College Hospitals (IHEC/SDC/OMFS/2204/23/155). Informed consent was obtained from all the participants of this study. Patients in the age range of 20-35 years who underwent mandibular third molar surgery were recruited for the study. Patients who were allergic to local anesthetics, presence of infection and inflammation at the site of needle insertion, and any systemic conditions that may affect anesthesia outcomes were excluded from the study.

Using the G*Power software (program written by Franz Faul, Universität Kiel, Germany), analysis was performed to determine the sample size required for the study considering the expected effect size, significance level, and power. A sample size of 100 was determined to achieve sufficient statistical power for detecting differences in achieving the onset of anesthesia and patient perception of pain. A total of 100 participants were randomly assigned to five different groups representing various IANB techniques with 20 patients in each group. The five IANB techniques followed in this study are the conventional Halsted technique, Vazirani-Akinosi technique, Gow-Gates technique, Fischer 1-2-3 technique, and extraoral (Kurt-Thoma) technique. Randomization was performed using computer-generated random numbers to ensure equal distribution of participants across the five groups. Under local anesthesia, surgical removal of the impacted mandibular third molar teeth was done, suturing was completed, and postoperative medications and instructions were given.

Data were collected by trained researchers and dental professionals who were blinded to the assigned techniques. Information regarding demographic characteristics, medical history, and baseline data were recorded. The onset of anesthesia and patients' perception of pain were documented for each technique, and any adverse events or complications were also recorded.

Conventional Halsted technique

**Figure 1:**
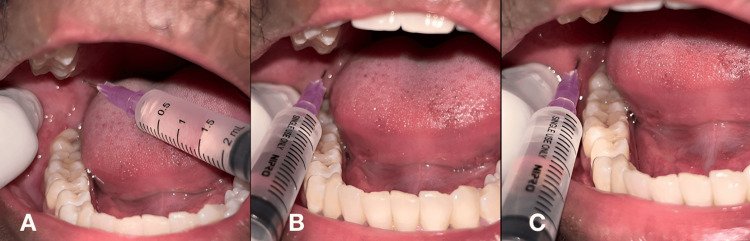
Conventional Halsted technique A. inferior alveolar nerve block; B. lingual nerve block; C. long buccal nerve block

The clinical landmarks for this technique are the pterygomandibular raphe, coronoid notch, and occlusal surface of the mandibular molar. Initially, the coronoid notch was palpated. The syringe barrel was placed on the contralateral premolar, and a needle was advanced over a depth of 18-25 mm until the bony contact was felt. Aspiration was done, and 1.2 ml of a local anesthetic solution was deposited for anesthetizing the inferior alveolar nerve. The needle was turned to the ipsilateral side, and 0.5 ml of the solution was deposited to anesthetize the lingual nerve. To anesthetize the long buccal nerve, 0.3 ml of the local anesthetic was deposited distal to the distal molar [8]. The technique is depicted in Figure 1.

Fischer 1-2-3 technique

**Figure 2:**
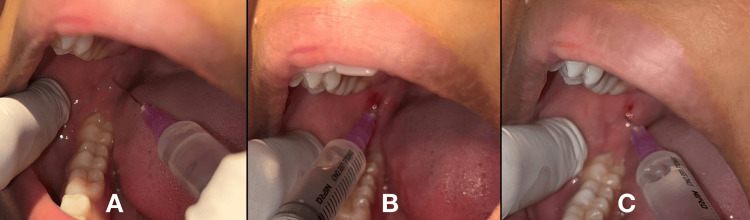
Fischer 1-2-3 technique A. long buccal nerve block; B. lingual nerve block; C. inferior alveolar nerve block

The anatomical landmarks were palpated first, the needle was inserted distal to that of the distal molar, and a local anesthetic solution of 0.5 ml was deposited to anesthetize the long buccal nerve. Later, the syringe was positioned on the occlusal surface of opposing premolars, and the needle was placed six millimeters deep at the spot where guide fingernails meet at the external oblique ridge. The needle was inserted, and after attaining a bony contact, 1.2 ml of the solution was deposited, and the needle was turned to the ipsilateral side and halfway retracted, following which 0.5 ml of the solution was deposited [9]. This technique is depicted in Figure 2.

Vazirani-Akinosi technique

**Figure 3:**
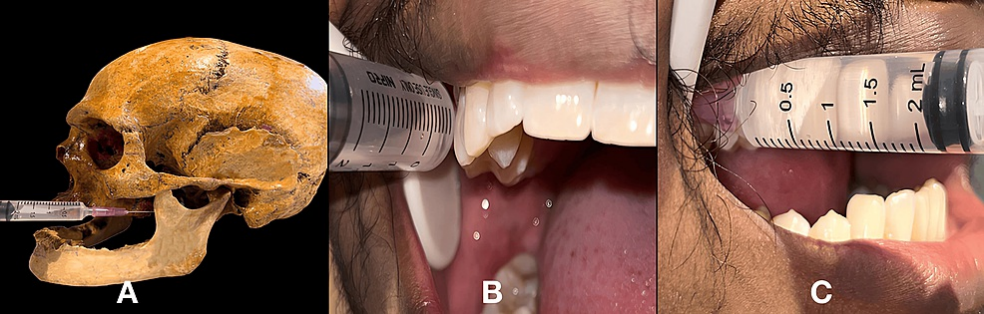
Depicting the Vazirani-Akinosi technique. A. depicting the Vazirani-Akinosi technique on a skull model; B and C. depicting the technique in a patient

The patients were asked to close their mouths in order to place the needle parallel to the occlusal plane. The needle was introduced up to 1.5 inches medial to the ramus while keeping the syringe at the level of the mucogingival junction of the maxillary molars. After several aspirations, 1.2 ml of the solution was deposited. It had advantages over the traditional open-mouth method because landmarks were easier to identify, and the entire area was anesthetized with a single injection [10]. This technique is depicted in Figure 3.

Gow-Gates technique

**Figure 4:**
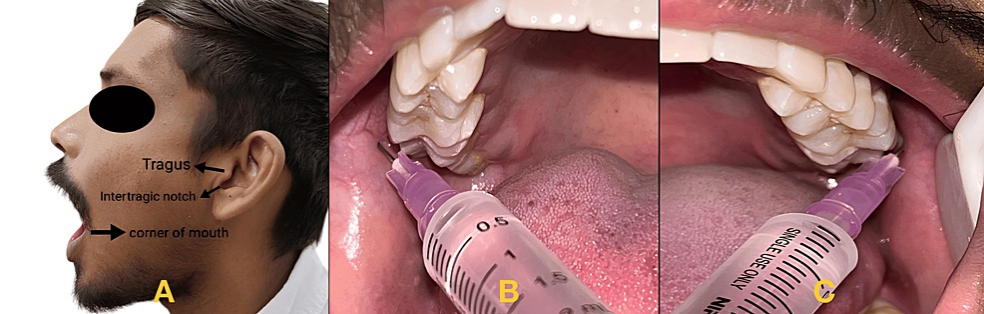
Gow-Gates technique A. extraoral landmarks for the Gow-Gates technique; B and C depicting the point of entry for the Gow-Gates technique

By palpating the external oblique ridge of the anterior surface of the ramus in the coronoid notch, the bony landmark was identified intraorally. The barrel of the syringe was positioned on the contralateral premolar or canine. The needle tip was aimed for the neck of the condyle, and the patient was asked to open their mouth as widely as possible. For this strategy to be successful, a large opening is absolutely necessary. The needle was advanced deeper until a bony contact was made. This contact was made at a depth of 25 mm, although individuals with a noticeably flared ramus needed a deeper touch. Once a bony contact was made, the needle was withdrawn for 1 mm, and 1.8 ml of the anesthetic solution was deposited [11]. This technique is depicted in Figure 4.

Extraoral technique by Kurt and Thoma

**Figure 5:**
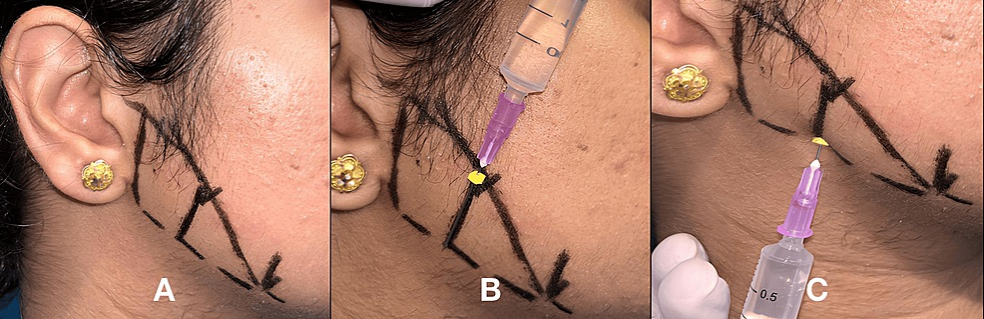
Extraoral Kurt-Thoma technique A. depicting the markings for the Kurt-Thoma technique; B. a rubber stopper was placed for the needle and the length of insertion was measured; C. insertion of the needle at the inferior border of the mandible

Prior to injecting, the skin was well cleaned. First, the patient was asked to clench their teeth, and then the lower-most anterior part of the masseter muscle was marked. From this point to the tragus of the ear, a line was drawn. A midpoint was marked, and from this midpoint, a second line parallel to the back of the mandible was drawn and measured. The needle had the same length markings as the measurements. From the lower border of the mandible toward the medial side, a long needle was passed as close to the bone as feasible, up to the mark, and a solution of 1.2 ml was slowly administered [12]. This technique is depicted in Figure 5.

Outcome measures

The outcome measures were the onset of anesthesia, pain perception during local anesthesia administration, and trismus. The onset of anesthesia was the time elapsed from the deposition of the local anesthesia till the subjective and objective symptoms were positive. Subjective assessments by the patients themselves were recorded at regular intervals by questioning the patient. In the objective method after administration of local anesthesia, using the electric pulp tester, the pulpal response was noted. Absence of pulpal response at 10 on the electric pulp tester was considered a positive objective symptom. Pain perception during local anesthesia administration was evaluated using VAS scores. The patients were asked to rate their pain experience during anesthesia administration. Incidence of postoperative trismus was noted, and patients with limited mouth opening of less than 30 mm were considered to have trismus.

Statistical analysis

Data were analyzed using IBM SPSS Statistics for Windows, version 23.0 (released 2015; IBM Corp Armonk, United States), with p-values less than 0.05 considered as statistically significant. Descriptive statistics, Kruskal-Wallis, and post-hoc tests were included in the data analysis for intergroup comparisons.

## Results

Our study consisted of 100 participants with 52 male patients and 48 female patients. Patients with a mean age range of 26 ± 2.5 years were enrolled in this study and split into five groups, with 20 participants in each group. 

Onset of anesthesia

**Figure 6:**
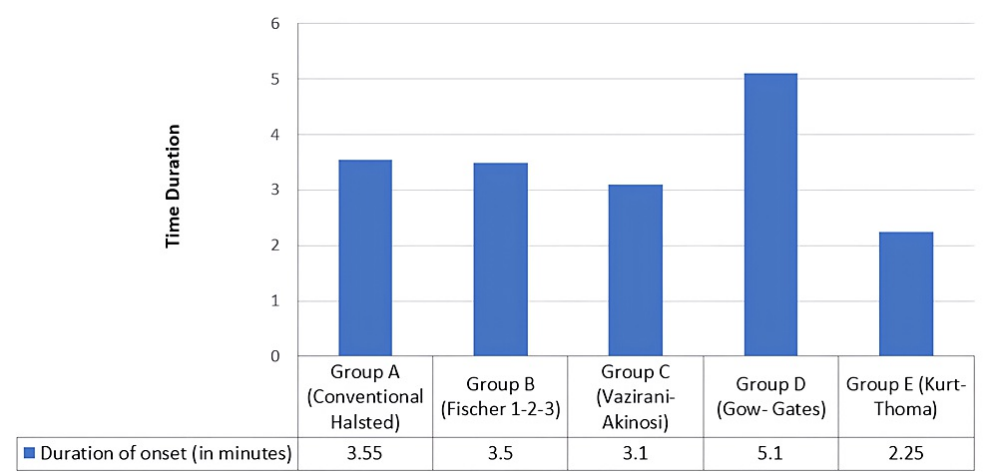
Mean onset of anesthesia among the different groups

The onset of anesthesia was measured from the time of deposition of local anesthesia till the subjective and objective signs were attained. The mean onset of anesthesia in different techniques of the IANB is depicted in Table 1 and Figure 6.

**Table 1:** Mean onset of anesthesia among the different groups The mean duration of the onset of anesthesia was faster in the Kurt-Thoma technique, with a mean duration of 2.25 minutes.

Group	Onset of anesthesia				
	N	Minimum (minutes)	Maximum (minutes)	Mean (minutes)	Standard deviation
Group A (conventional Halsted)	20	3	4	3.55	0.510
Group B (Fischer 1-2-3)	20	3	5	3.50	0.607
Group C (Vazirani-Akinosi)	20	2	4	3.10	0.553
Group D (Gow-Gates)	20	4	6	5.10	0.718
Group E (Kurt-Thoma)	20	2	3	2.25	0.444

Perception of pain

**Figure 7:**
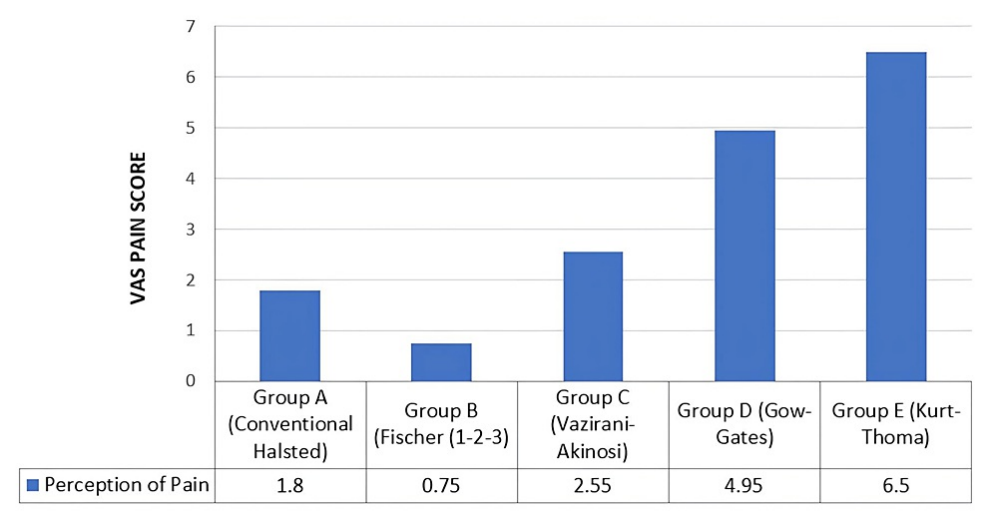
Mean pain score during the administration of local anesthesia among the different groups

Perception of pain during the administration of the local anesthesia was measured using the 10-point VAS. It was noted that perception of pain was higher in group E (Kurt-Thoma techniqure) compared to that of the other groups. The results are depicted in the 2 and Figure 7.

**Table 2:** Mean pain score during the administration of local anesthesia among the different groups Pain during the administration of the local anesthesia was the highest with the Kurt-Thoma technique, and it was the lowest with the Fischer 1-2-3 technique.

Group	Perception of pain				
	N	Minimum	Maximum	Mean	Standard deviation
Group A (Conventional Halsted)	20	0	2	1.80	0.523
Group B (Fischer 1-2-3)	20	0	1	.75	0.444
Group C (Vazirani-Akinosi)	20	2	4	2.55	0.605
Group D (Gow-Gates)	20	4	6	4.95	0.510
Group E (Kurt-Thoma)	20	5	7	6.50	0.761

Incidence of trismus

**Figure 8:**
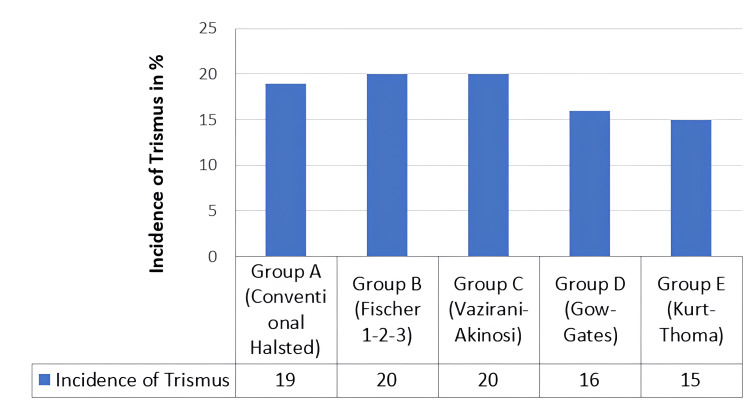
Incidence of trismus among the different groups

The incidence of trismus was noted to be higher in Group E compared to that of the other groups, and the results are depicted in Table 3 and Figure 8.

**Table 3:** Incidence of trismus among the different groups The incidence of trismus was highest with the Kurt-Thoma technique.

Group	Frequency	
	Absent	Present
Group A (Conventional Halsted)	19 (95%)	1 (5%)
Group B (Fischer 1-2-3)	20 (100%)	0(0%)
Group C (Vazirani-Akinosi)	20 (100%)	0(0%)
Group D (Gow-Gates)	16 (80%)	4 (20%)
Group E (Kurt-Thoma)	15 (75%)	5 (25%)

Comparison of the onset of anesthesia in the different groups

The inspection of the quantile-quantile (QQ) plot and Shapiro-Wilk test revealed that the duration of the onset of anesthesia was not normally distributed for all the groups. Therefore, Kruskal-Wallis test was run on the data keeping the significance level at 0.05, which is depicted in Table 4.

**Table 4:** Comparison of the onset of anesthesia among the different groups *p = 0.000 - statistically significant; * IQR - interquartile range; * df - degrees of freedom

Group	Onset of anesthesia						Test statistics	df	p-value
	N	Minimum	Maximum	Mean rank	Median	IQR			
Group A (Conventional Halsted)	20	3	4	55.15	4	1	46.834	4	0.000
Group B (Fischer 1-2-3)	20	3	5	52.80	3	1			
Group C (Vazirani-Akinosi)	20	2	4	40.80	3	0			
Group D (Gow-Gates)	20	4	6	87.75	5	1			
Group E (Kurt-Thoma)	20	2	3	16.00	2	1			

The test revealed a statistically significant difference in the duration of the onset of anesthesia between the groups (x^2^(4) = 46.834, p = 0.000). Therefore, a post-hoc test for pairwise comparison was performed, which is depicted in Table 5. 

**Table 5:** Intergroup comparison of onset of anesthesia ** statistically significant

Duration of the onset of anesthesia		Test statistics	p-value
Sample 1	Sample 2		
Group E (Kurt-Thoma)	Group C (Vazirani-Akinosi)			24.80	0.047**
Group E (Kurt-Thoma)	Group B (Fischer 1-2-3)	36.80	0.000**
Group E (Kurt-Thoma)	Group A (Conventional Halsted)	39.15	0.000**
Group E (Kurt-Thoma)	Group D (Gow-Gates)	71.75	0.000**
Group C (Vazirani-Akinosi)	Group B (Fischer 1-2-3)	12.00	1.000
Group C (Vazirani-Akinosi)	Group A (Conventional Halsted)	14.35	1.000
Group C (Vazirani-Akinosi)	Group D (Gow-Gates)	.- 46.950	0.000**
Group B (Fischer 123)	Group A (Conventional Halsted)	2.35	1.000
Group B (Fischer 123)	Group D (Gow Gates)	-34.950	0.001**
Group A (Conventional Halstedl)	Group D (Gow Gates)	-32.60	0.002**

On comparing the different groups for the duration of the onset of anesthesia, the following results were obtained: There was a statistically significant difference between Group E (Kurt-Thoma) when compared to the other groups, i.e., Group C (Vazirani-Akinosi) (p = .047), Group A (conventional Halsted) (p = 0.000), and Group D (Gow-Gates) (p = 0.000). These results conclude that the onset of anesthesia was faster with the Kurt-Thoma technique compared to that of the other techniques.

Comparison of the perception of pain in different groups

The inspection of the QQ plot and Shapiro-Wilk test revealed that the perception of pain was not normally distributed for all the groups. Therefore, a Kruskal-Wallis test was run on the data with the significance level set at 0.05, which is depicted in Table 6.

**Table 6:** Comparison of pain perception during the administration of local anesthesia among different groups p = 0.000 - statistically significant

Group	Perception of pain						Test statistics	df	p-value
	N	Minimum	Maximum	Mean rank	Median	IQR			
Group A (conventional Halsted)	20	3	4	33.13	2	0	80.000	4	0.000
Group B (Fischer 1-2-3)	20	3	5	12.13	1	1			
Group C (Vazirani-Akinosi)	20	2	4	46.33	2.50	1			
Group D (Gow-Gates)	20	4	6	72.05	5	0			
Group E (Kurt-Thoma)	20	2	3	88.88	7	1			

The test revealed that there is a statistically significant difference in the perception of pain between the groups (X^2^(4) = 80.00, p = 0.000). Therefore, a post-hoc test for pairwise comparison was performed, as shown in Table 7.

**Table 7:** Intergroup comparison of patient perception of pain during administration of local anesthesia ** statistically significant

Perception of pain		Test statistics	p-value
Sample 1	Sample 2		
Group B (Fischer 1-2-3)	Group A (Conventional)			21.00	0.199
Group B (Fischer 1-2-3)	Group C (Vazirani-Akinosi)	-34.20	0.001**
Group B (Fischer 1-2-3)	Group D (Gow-Gates)	-59.925	0.000**
Group B (Fischer 1-2-3)	Group E (Kurt-Thoma)	-76.750	0.000**
Group A (Conventional Halstedl)	Group C (Vazirani-Akinosi)	-13.200	1.000
Group A (Conventional Halstedl)	Group D (Gow-Gates)	-38.925	0.000**
Group A (Conventional Halsted)	Group E (Kurt-Thoma)	-55.750	0.000**
Group C (Vazirani-Akinosi)	Group D (Gow-Gates)	-25.725	0.043**
Group C (Vazirani-Akinosi)	Group E (Kurt-Thoma)	-42.550	0.000**
Group D (Gow-Gates)	Group E (Kurt-Thoma)	-16.825	0.621

On comparing the different groups for patients' perception of pain during the administration of anesthesia, the following results were obtained: There was a statistically significant difference between Group B (Fischer 1-2-3) and Group C (Vazirani-Akinosi) (p = 0.001), Group D (Gow-Gates) (p = 0.000), and Group E (Kurt-Thoma) (p = 0.000), with mean ranks of 46.33, 72.05, and 88.88, respectively, in the perception of pain compared with that of Group B with a mean rank of 12.13. These results conclude that patients' perception of pain during the administration of local anesthesia was minimal with the Fischer 1-2-3 technique.

Comparison of incidence of trismus in the different groups

Based on the results of the chi-square test, it can be stated that there was a significant association between the groups and the incidence of trismus (X2-12.22, p = 0.016), with Group E (5) showing the highest incidence of trismus, followed by Group D (4), Group A (1), Group B (0), and Group C (0), which is depicted in Table 8.

**Table 8:** Comparison of the incidence of trismus among the different groups ** statistically significant

Group	Frequency of the incidence of trismus		Pearson chi-square	df	p-value
	Absent	Present			
Group A (Conventional Halsted)	19 (95%)	1 (5%)	12.222	4	0.016**
Group B (Fischer 1-2-3)	20 (100%)	0(0%)			
Group C (Vazirani-Akinosi)	20 (100%)	0(0%)			
Group D (Gow-Gates)	16 (80%)	4 (20%)			
Group E (Kurt-Thoma)	15 (75%)	5 (25%)			

## Discussion

Due to the greater density of the mandibular alveolar bone, restricted access to the inferior alveolar nerve, anatomical variations, and the requirement for deeper needle penetration into the soft tissues, the anesthetic techniques for mandibular structures have a lower success rate than those for maxillary structures [8-10]. To overcome these problems and to achieve profound anesthesia, various techniques of IANBs came into existence, including the conventional Halsted technique, Vazirani-Akinosi technique, Gow-Gates technique, Fischer 1-2-3 technique, and extraoral (Kurt-Thoma) technique.

William S. Halsted and Richard J. Hall administered a cocaine solution near the mandibular foramen at the end of November 1884 to establish the first neuroregional anesthesia in the mandible [11]. In the year of 1966, Angelo Sargenti gave the first modification to the conventional Halsted technique in which the positioning of the needle was at a higher level compared to that of the conventional technique [12]. In the direct thrust technique, the coronoid notch is palpated, and the index finger is used as a guide. An imaginary line is extended from the index finger, and the needle is inserted into the pterygomandibular raphe, and after attaining a bony contact, the solution is deposited. This technique was described by Dr. Mendel Nevin and was further modified by Dr. Borris Levill and Dr. I.R. Brownle in which the needle is penetrated at a point midway between the maxillary and mandibular occlusal planes [13].

In Clarke and Holmes' technique, a local anesthetic solution is deposited behind the mandibular foramen, as the anterior part of the foramen has two important structures, i.e., lingula and attachment of the sphenomandibular ligament. However, most fibers of the anterior were not anesthetized, which was the main drawback of their technique [14]. The Vazirani-Akinosi technique is especially applicable in patients with limited mouth opening. The Gow-Gates technique is not easy to perform by new practitioners as this technique is highly technique-sensitive and could be performed only if a wide mouth opening is possible [14]. The Fischer 1-2-3 technique is similar to that of the conventional technique with the exception that the long buccal nerve is anesthetized first, followed by the lingual nerve and inferior alveolar nerve. Although the Kurt-Thoma technique has adequate landmarks and depicts the location of the mandibular foramen accurately, the anxiety of the patient is highest compared to that of the other techniques, because it is an extraoral approach [15].

In our study, we excluded patients with a history of allergy to local anesthetics, infections, and inflammation at the site of the needle insertion and patients with systemic conditions as these factors will affect the outcome of the results. No research was made, and there was no evidence in comparing various techniques of the IANB in the published literature [2,5,16].

The results of this prospective study have shown that the Kurt-Thoma technique had the fastest onset of anesthesia (2.25 minutes) compared to that of other techniques, although pain perception was the highest with a score of 6.5 in the VAS and presented with the highest incidence of trismus (25%). The rapid onset of action in this technique can be attributed to the deposition of the local anesthetic solution close to the site of the lingula [6]. Although it had a rapid onset of action, it had drawbacks of higher patient perception of pain and trismus, and as it was an extraoral approach, the patient's anxiety levels were higher.

The Gow-Gates technique had the slowest onset of anesthesia (5.1 minutes) compared to the other techniques as the solution is deposited at the condyle region, and it also takes a prolonged duration of time to anesthetize the core fibers in the proximal segment of the inferior alveolar nerve [9]. Apart from this, the procedure is technique-sensitive, and it will be difficult for new beginners. It also deems wide opening of the mouth of the patients and is difficult to locate the anatomical landmarks for this technique compared to the other techniques [10].

In our study, the conventional Halsted technique, Fischer 1-2-3 technique, and Vazirani-Akinosi technique have shown an almost similar duration for the onset of anesthesia with 3.55, 3.5, 3.1 minutes, respectively. Patients' perception of pain during the administration of the local anesthetic was the least with the Fischer 1-2-3 technique with a VAS score of 0.75 and highest in the Kurt-Thoma technique with a 6.5 VAS score, followed by the Gow-Gates technique with a 4.95 VAS score. The incidence of trismus was higher with the Kurt-Thoma technique (25%), followed by the Gow-Gates technique (20%) and least in the conventional Halsted technique (5%).

The advantage of the Fischer 1-2-3 technique will be less pain perception to the patient as sequential blocking of the nerves happens with this technique, i.e., long buccal nerve, lingual nerve, and inferior alveolar nerve [17]. The Vazirani-Akinosi technique is useful, especially in patients with limited mouth opening. Although the patients' pain perception was minimal with this technique, there are no adequate landmarks for this procedure [18].

The conventional Halsted technique is one of the easiest techniques to perform compared to all the other techniques as it is easier to locate the anatomical landmarks [19]. In our study, the conventional Halsted technique did not differ in terms of onset of anesthesia and pain perception during administration of local anesthesia when compared to that of the Fischer 1-2-3 technique. Thus, the conventional Halsted technique remains the gold standard technique. These results may aid dentists in selecting the most suitable technique based on patient preferences and procedural requirements.

 Limitations of the study

This study was conducted on a small population, and it has to be performed on a larger sample size to improve the accuracy of the data. The study was conducted in the population of Tamil Nadu, and it is a single-center study, so further studies have to be conducted at multicenter level.

## Conclusions

In this study, it was found that the conventional Halsted technique was the best among the different techniques of IANB and remains the gold standard. All these findings contribute to improving the clinical practice of dental anesthesia, enhancing patient satisfaction, and optimizing treatment outcomes. Choosing the appropriate anesthesia technique has significant implications in clinical practice, benefiting both oral surgeons and patients.
